# The short-term and long-term effects of intranasal mesenchymal stem cell administration to noninflamed mice lung

**DOI:** 10.3389/fimmu.2022.967487

**Published:** 2022-09-16

**Authors:** Marlena Tynecka, Adrian Janucik, Magdalena Niemira, Arkadiusz Zbikowski, Nino Stocker, Agnieszka Tarasik, Aleksandra Starosz, Kamil Grubczak, Anna Szalkowska, Urszula Korotko, Joanna Reszec, Miroslaw Kwasniewski, Adam Kretowski, Cezmi Akdis, Milena Sokolowska, Marcin Moniuszko, Andrzej Eljaszewicz

**Affiliations:** ^1^ Department of Regenerative Medicine and Immune Regulation, Medical University of Bialystok, Bialystok, Poland; ^2^ Clinical Research Centre, Medical University of Bialystok, Bialystok, Poland; ^3^ Department of Medical Biology, Medical University of Bialystok, Bialystok, Poland; ^4^ Swiss Institute of Allergy and Asthma Research, University of Zurich, Davos, Switzerland; ^5^ Department of Medical Pathomorphology, Medical University of Bialystok, Bialystok, Poland; ^6^ Centre for Bioinformatics and Data Analysis, Medical University of Bialystok, Bialystok, Poland; ^7^ Department of Endocrinology, Diabetology and Internal Medicine, Medical University of Bialystok, Bialystok, Poland; ^8^ Department of Allergology and Internal Medicine, Medical University of Bialystok, Bialystok, Poland

**Keywords:** mesenchymal stem cell, noninflamed lung, stem cell-based therapy, epithelial barrier, transcriptomic profiles

## Abstract

Mesenchymal stem cells (mesenchymal stromal cells; MSC)-based therapies remain a promising approach to treat degenerative and inflammatory diseases. Their beneficial effects were confirmed in numerous experimental models and clinical trials. However, safety issues concerning MSCs’ stability and their long-term effects limit their implementation in clinical practice, including treatment of respiratory diseases such as asthma, chronic obstructive pulmonary disease, and COVID-19. Here, we aimed to investigate the safety of intranasal application of human adipose tissue-derived MSCs in a preclinical experimental mice model and elucidate their effects on the lungs. We assessed short-term (two days) and long-term (nine days) effects of MSCs administration on lung morphology, immune responses, epithelial barrier function, and transcriptomic profiles. We observed an increased frequency of IFNγ- producing T cells and a decrease in occludin and claudin 3 as a long-term effect of MSCs administration. We also found changes in the lung transcriptomic profiles, reflecting redox imbalance and hypoxia signaling pathway. Additionally, we found dysregulation in genes clustered in pattern recognition receptors, macrophage activation, oxidative stress, and phagocytosis. Our results suggest that *i.n.* MSCs administration to noninflamed healthy lungs induces, in the late stages, low-grade inflammatory responses aiming at the clearance of MSCs graft.

## Introduction

Since the 90s mesenchymal stem cells (also known as mesenchymal stromal cells, MSCs) have gained considerable interest in the scientific community due to their immunomodulatory properties and regenerative potential. To date, the MSC-mediated beneficial effect has been confirmed in numerous preclinical models, medical experiments, and clinical trials (1–5). In fact, the perspective of MSC-based therapy implementation holds a promise for, to date, uncurable or poorly controlled chronic inflammatory and degenerative diseases. According to *clinicatrial.gov*, MSCs have been tested in multiple respiratory system diseases, such as acute respiratory distress syndrome, COVID-19, chronic obstructive pulmonary disease, idiopathic pulmonary fibrosis, and asthma.

MSCs are adult multipotent stem cells, initially identified and isolated from the bone marrow (6, 7). Currently, they are replaced by counterparts derived from more accessible sources, such as adipose tissue, Wharton’s jelly, and cord blood (2, 5, 8–12).

Notably, differentially sourced MSCs may slightly differ in functional properties and phenotype. Therefore, minimal criteria for their definition have been proposed, namely: i) plastic adherence; ii) surface expression of CD29, CD71, CD73, CD90, CD105, CD271, and simultaneous lack of CD14, CD34, CD45, and HLA-DR; iii) ability to differentiate into at least osteoblasts, adipocytes, and chondrocytes *in vitro* (1). Accumulating evidence shows that their immunoregulatory properties need to be activated by the pro-inflammatory microenvironment. Thus, MSC’s anti-inflammatory potential depends on the local milieu. MSCs have been shown to exert immunoregulatory function by i) reduction of monocyte and CD34^+^ cell maturation towards classically activated pro-inflammatory M1 macrophages and dendritic cells (DCs); ii) reduction of adaptive immune responses; iii) recruitment of regulatory T cells (Treg) and induction of effector T cell plasticity towards anti-inflammatory properties; and iv) reduction of cytotoxic innate lymphoid cell activity (1, 8, 13). Unfortunately, MSCs fate in non-inflamed tissue remains elusive.

To date, safety issues concerning the long-term effects of MSCs administration and their stability are raised, limiting their usage in the clinical practice (1, 2, 14). To better understand the effects of MSCs administration on the airway microenvironment, here we aimed to investigate the longitudinal changes in the lung morphology, epithelial barrier function, immune responses, and transcriptomic profiles of the normal non-inflamed lung in the mouse model. In our model, we used MSCs derived from adipose tissue, representing an attractive and highly available source of these cells.

## Materials and methods

### Experimental mouse model

**Figure 1:**
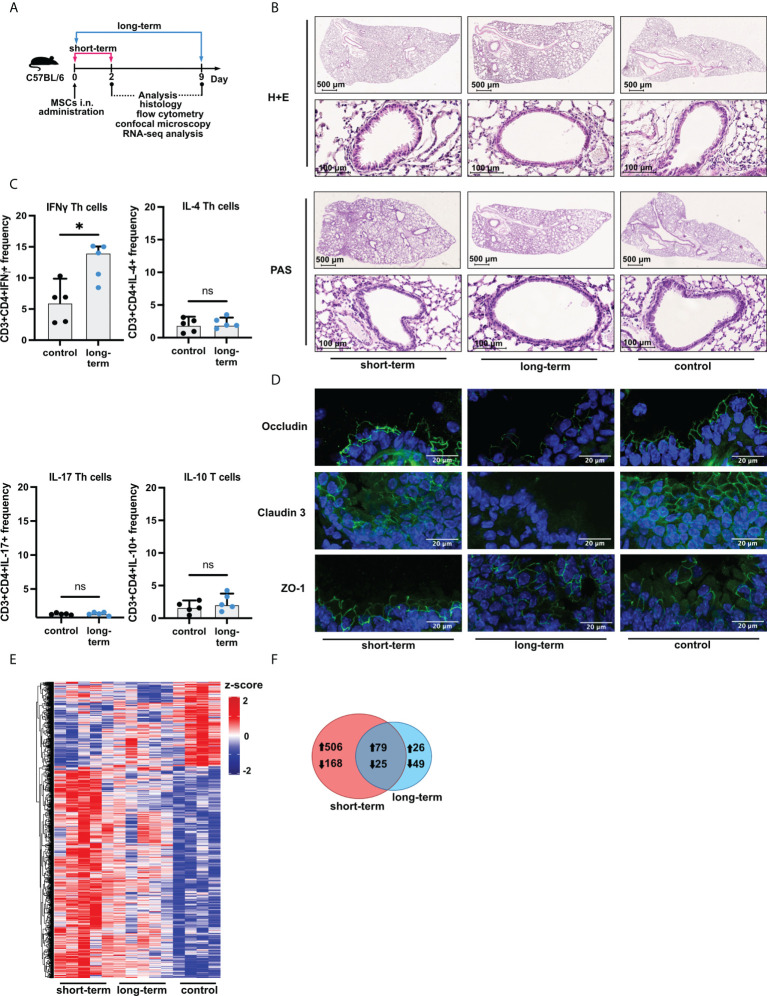
Induction of low-grade inflammation is a long-term effect of intranasal administration of adipose-tissue-derived mesenchymal stem cells. **(A)** Experimental mice model used in the study. Female C57BL/6 mice were sacrificed directly after two days (short-term), and nine days (long-term) after mesenchymal stem cells (MSCs) intranasal (*i.n.*) administration. Saline (vehicle)-treated mice were used as control. **(B)** Representative Hematoxylin & Eosin (H&E) and Periodic Acid-Shiff (PAS) staining in the lung sections. **(C)** Summary of flow cytometry analyses of IFNγ, IL-4, IL-17, or IL-10 producing T cell frequency after MSCs administration in the long-term model. U Mann-Whitney test was used to evaluate differences between groups, *p < 0.05; ns, not significant; n=5. **(D)** Representative confocal staining of occludin, claudin 3, and zonula occludens-1 (ZO-1) in the alveoli after MSCs *i.n.* administration; DAPI – blue; green – positive signal for analyzed proteins. **(E)** Summary of transcriptomic profiles of the lung after MSCs *i.n.* administration. The heatmap shows all (n =853) differentially regulated genes (DEGs) among the analyzed groups. DEGs were identified based on |Log2FoldChange| > 0.5, and adjusted p value < 0.1. Cutoffs were applied with matched HGNC identifiers. Complete linkage clustering was applied. **(F)** Venn diagram of differentially and commonly regulated genes in the short- (marked red) and long-term model (marked blue). Arrows indicate the up- or down-regulated expression of DEGs.

Female 6-8-week-old C57BL/6 mice were divided into three groups (n=5 per group). Mice were sacrificed after 2 (short-term) and 9 (long-term) days after intranasal (*i.n*.) administration of the adipose-tissue-derived MSCs (**Figure 1A**; for detailed method description please see **Supplementary Materials – methods**). Biological material was collected and biobanked for further analyses.

### Histochemical staining’s

The presence of inflammatory infiltration and mucus production in the lung was assessed by histochemical stainings. First, lungs were fixed in 4% paraformaldehyde and paraffin-embedded. Next, 4μm microtome sections were placed on the glass slides (Thermofisher Scientific) and stained with hematoxylin-eosin (H+E) and Periodic acid-Shiff (PAS) according to the standard protocols. The slides were visualized using a digital slide scanner Nanozoomer SQ (Hamamatsu). Both H+E and PAS staining’s were quantified in ImageJ software.

### Quantification of hematoxylin-eosin (H+E) and periodic acid-shiff (PAS) staining

Inflammatory infiltration within the lung tissue was quantified using ImageJ software (NIH) in H+E-stained slides. The default thresholding method and the HSB model for color space were selected to perform the analysis. The lung tissue surface was measured using a threshold tool. The slider in the brightness panel was appropriately adjusted to cover all tissue areas. Additionally, to evaluate the inflammation surface the slider in the hue panel was acquired to imply the dark pink-purple colors. Three independent measurements were performed and the mean was calculated. The results were presented as inflammation area to tissue (slide) area ratio. Additionally, all the values were normalized to the mean of control group measurements. To evaluate the mucus production in PAS-stained slides, the quantification was restricted only to bronchi. To maximize the relevance of the results two different bronchioles within one tissue slide were taken into consideration. Similarly, to assess the mucus area, the slider was adjusted to incorporate pink-red colors. Additionally, the surface inside all bronchi was measured. To simplify the calculations, the bronchi shape was assumed as a circle and the perimeter was calculated. Three measurements for all bronchioles were performed and the mean was calculated. The results were presented as a ratio of mucus area and bronchioles perimeter score.

### Flow cytometry

Lung tissue dissociation was performed using Lung Dissociation Kit (Miltenyi Biotec). Next, cells were stimulated with Leukocyte Activation Cocktail with Golgi Plug (BD Pharmingen) for 3 hours. Extracellular and intracellular staining was performed according to the standard protocol using a panel of fluorochrome-labeled monoclonal antibodies (**Supplementary Materials – Supplementary Table S2**). Cells were acquired using the FACSAria system (BD Biosciences) and analyzed with the FlowJo v.10 software (BD Biosciences). A representative gating strategy has been presented in the **Supplementary Materials – Supplementary Figure S2A**.

### Immunofluorescence staining

Snap-frozen lung tissues were cryosectioned (Leica CM3050 S) at 8μm, and subsequently fixed with 4% paraformaldehyde. Cryosections were submerged in blocking solution (10% goat serum, 1% bovine serum albumin, and 0,2% TritionX100) prior to incubation with polyclonal rabbit anti- ZO- 1 antibody (Invitrogen), monoclonal mouse anti-occludin (Invitrogen) at 1:200, and polyclonal rabbit anti-claudin 3 (Invitrogen) at 1:100 in 1% BSA in PBS, followed by incubation with Alexa Fluor 488 Goat anti-Rabbit IgG (Invitrogen) or Goat anti-Mouse IgG (Invitrogen) at 1:1000. Specimens were analyzed using a Zeiss LSM780 microscope (Zeiss). The detailed information on used antibodies has been presented in the **Supplementary Materials – Supplementary Table S2**.

### Statistical analysis

Statistical analysis was performed using GraphPad Prism v.9. Statistical significance was evaluated by the U Mann-Whitney test; p<0.05 was considered significant.

### RNA isolation and next-generation sequencing (NGS)

Lung lobes were stored in RNA later solution (Invitrogen) for 48 hours to stabilize RNA. Next, tissues were disrupted using TissueRuptor II (Qiagen) in RNeasy Lysis Buffer (Qiagen). Total RNA was isolated by using the RNeasy Mini Kit according to the manufacturer’s protocol (Qiagen). 1 µg of total RNA with RNA integrity number (RIN) > 8, was subjected to the cDNA library preparation according to TruSeq Stranded Total RNA protocol (Illumina), followed by the quality confirmation by TapeStation 2200 (Agilent, CA, USA). Next-generation sequencing (RNAseq) was performed using the Illumina HiSeq 4000 platform generating 150 bp paired-end reads (2 x 75 bp). Subsequently, transcriptomic profiling and analysis were performed. The entire data set has been submitted to the NCBI GEO database: accession number GSE200028 (The datasets are currently private and available under access token: “ovivksgmxdgzhcz”, and will be released immediately after manuscript acceptance).

### Transcriptome profiling and analysis

Sequencing quality was evaluated by FastQC version 0.11.5. Reads were mapped to the reference genome of Mus musculus (GRCm38) using STAR aligner version 2.5.3a. The obtained read counts were used to differential expression analysis (http://www.bioinformatics.babraham.ac.uk/projects/fastqc/; https://qubeshub.org/resources/fastqc) (15). Differential gene expression analysis was performed using DESeq2 (16). To adjust the Wald test p-value, the procedure of Benjamini & Hochberg was applied. Additionally, the count matrix was transformed into Transcripts Per Milion (TPM) to normalize gene expression. Differentially expressed genes (DEGs) with matched HGNC symbols were identified based on adjusted p-values < 0.1, and absolute Log2FoldChange > 0.5. To visualize the expression of DEGs, Venn Diagram, Volcano Plots, and heatmap with complete linkage clustering was generated in “R”. Gene Set Enrichment Analysis was performed to reveal Gene Ontology terms present in the dataset. Subsequently, the top 20 terms according to the Benjamini & Hochberg adjusted *p*<0.05 were plotted on the graphs. Moreover, based on The Mouse Genome Database (MGD; http://www.informatics.jax.org; access date: 29^th^ June 2022), individual branches of Gene Ontology terms were selected for further analysis. Commonly regulated genes were analyzed using string. db and Ingenuity Pathway Analysis (IPA, QIAGEN Inc., https://digitalinsights.qiagen.com/IPA). The STRING gene networks were generated concerning all commonly regulated genes, including the predicted and RIKEN genes (n=104). Nodes without any connections were excluded from the network on set medium confidence levels. Using IPA generated pathways with altered z-score, commonly regulated genes for two investigated models were analyzed individually. A two-tailed Mann U Whitney test was applied to assess the difference in the expression of mentioned genes. The delta of the expression was presented using the R package ggplot2 (https://ggplot2.tidyverse.org). The expression datasets were analyzed in IPA with the cutoff points for absolute Log2FoldChange > 0.5 and adjusted p-value < 0.1 with additional lung tissue filters applied.

## Results

### Administration of the adipose tissue-derived mesenchymal stem cells induces low-grade inflammation and reduces epithelial barrier integrity

First, we aimed to confirm that adipose tissue-derived cells fulfill the criteria of mesenchymal stem cells established by the International Society for Cellular Therapy. We expanded plastic adherent cells and confirmed the surface expression of MSCs characteristic markers, namely CD73, CD90, and CD105, with simultaneous lack of lineage marker CD45 and human leukocyte antigen (HLA-DR) expression (**Supplementary Figure S1A**). Moreover, we successfully differentiated the cells *in vitro* into adipocytes, osteocytes, and chondrocytes (**Supplementary Figure S1B**).

Having confirmed that cells isolated from adipose tissue fulfill the criteria of MSCs, next, we wished to investigate the effects of their *i.n.* administration on the induction of inflammation in the lower airways (**Figure 1A**). We found no signs of increased cellular infiltration and mucus production within the lungs both directly (two days, short-term) and extendedly (nine days, long-term) after MSCs transfer (**Figure 1B**, **Supplementary Materials - Figure S2A**). However, we observed an increase in the frequency of INFγ producing, but not IL-4, IL-17, and IL-10 producing, CD3^+^CD4^+^ T cells in the lungs as an effect of the long-term MSCs administration (**Figure 1C**; **Supplementary Materials - Figure S2B**). Moreover, occludin and claudin 3, but not ZO-1 were decreased in the long-term model (**Figure 1D**). To better understand the effects of MSCs administration on the non-inflamed lungs, we next aimed to investigate MSC-mediated effects on the transcriptomic profiles of the lungs. We observed dynamic changes in the lung gene expression profiles after MSCs administration (**Figure 1E**). We found 674 differentially regulated genes unique for the short-term model, while only 75 genes were unique for the prolonged observations (**Figure 1F**), suggesting waning of the low-grade inflammation and active resolution in the longer time point. In addition, a total of 104 genes were common for both analyzed time points.

### Gene set enrichment analysis indicated changes in the activation of innate and adaptive immune responses after MSCs administration

**Figure 2:**
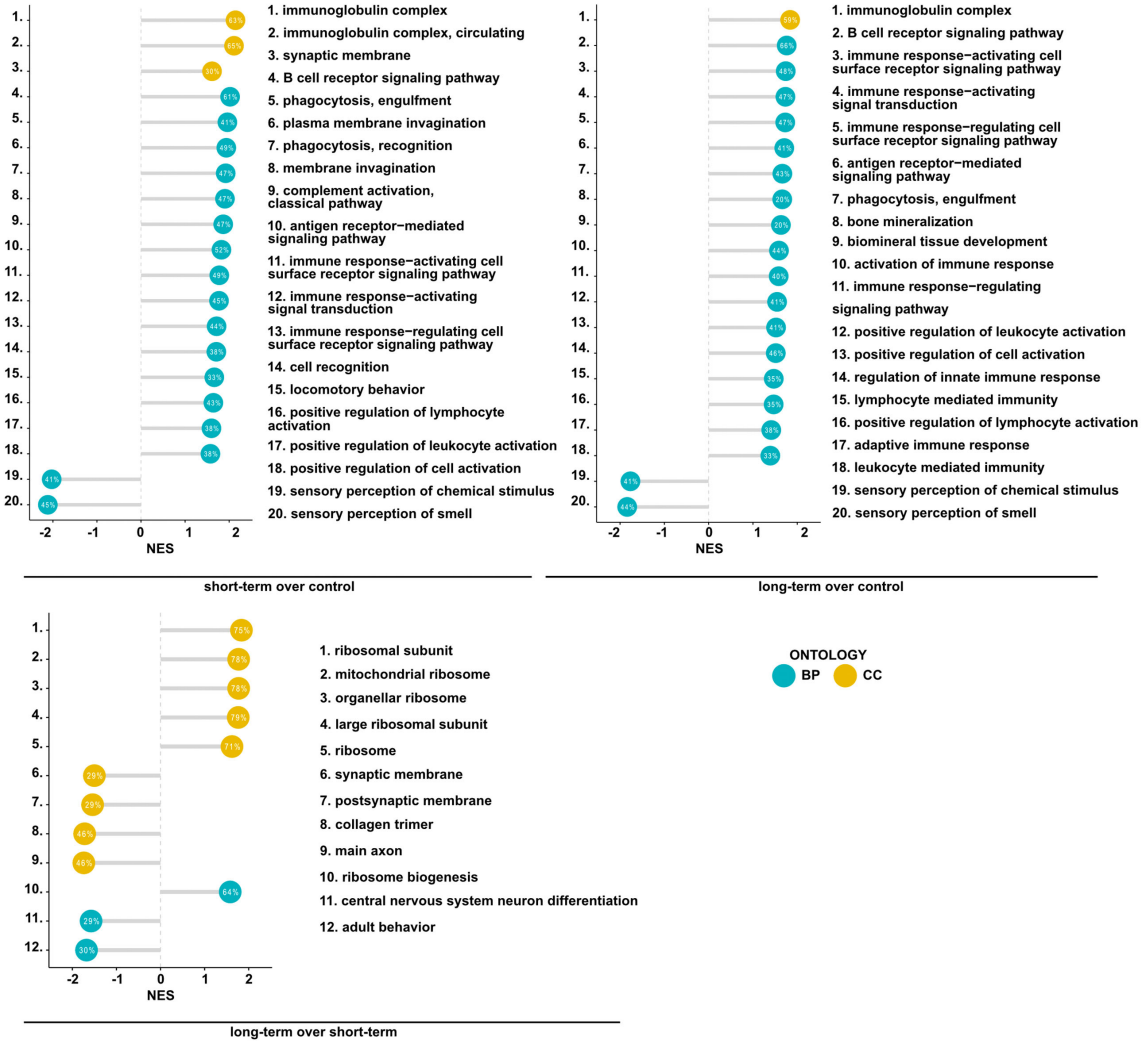
Mesenchymal stem cell administration to non-inflamed lungs induces the expression of genes associated with immunological pathways. Summary of gene set enrichment analysis for gene ontology. Top 20 most significant GO terms are listed according to the adjusted p-value. Ontologies are presented using lollipop charts with normalized enrichment scores. The percentage values represent the coverage of DEGs in each group to the theoretical size of an analyzed term. Blue and yellow refer to biological processes (BP) and cellular components (CC) of gene ontology, respectively.

**Figure 3:**
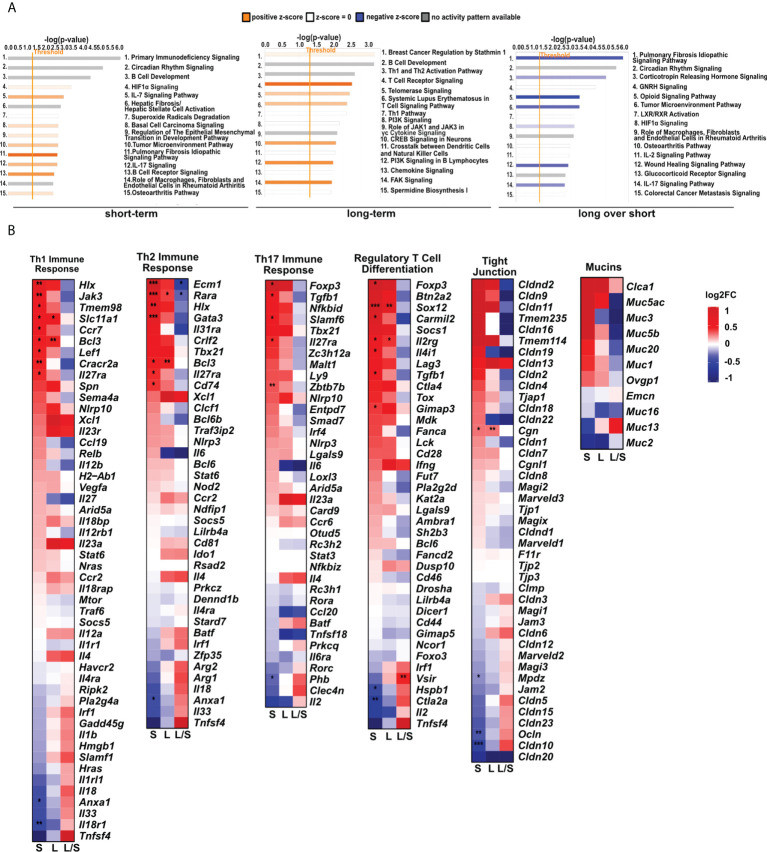
MSCs administration to noninflamed lungs causes changes in signaling pathways, and innate and adaptive immune gene clusters. **(A)** Changes in canonical and non-canonical signaling pathways after MSCs administration. A bar chart representing signaling pathways was generated using Ingenuity Pathway Analysis (IPA) Software. The top 15 most significant pathways in each group were presented. The gene cutoffs were adjusted on p-value<0.1, |Log2FoldChange|>0.5. Specific tissue filters restricting the analysis to pathways related to lungs were applied. Bars marked as red indicate pathway upregulation, while blue bars indicate downregulation. Grey bars refer to no activity pattern was available. White bars correspond to the pathways with a z-score = 0. **(B)** Heatmaps represent genes related to Th1-, Th2-, and Th17- driven immune response, differentiation of T regulatory cells, tight junction molecules, and mucins. The ratios considering significantly regulated genes to the total number of genes were equaled as follows: 11/67 for Th1- (Biological Process; GO:0042088), 8/62 for Th-2 (Biological Process; GO:0042092), 6/59 for Th17- driven immune response (Biological Process; GO:0072538), 9/42 for regulatory T cell differentiation (Biological Process; GO:0045066), 4/47 tight junctions (Tan et al. Allergy 2018), and 0/16 for mucins (Tan et al. Allergy 2018); S – short-term model; L – long-term model; L/S – long-term model *vs* short-term model; FDR<0.05; n=5; Wald test with Benjamini-Hochberg correction was used; *p<0.05; ** p<0.01; ***p<0.001.

Having found significant changes in the transcriptomic profiles, we next wished to elucidate whether differentially regulated genes may be functionally related, integrated, and referred to the specific genes clusters and interaction nodes. Thus, we evaluated the enrichment of DEGs in the gene ontologies, signaling pathways, and mapped predicted interactions using clusterProfiler v.4.0.0 (17). In the top 20 most significant gene ontology terms we found changes in immunological pathways. The normalized enrichment score (NES) analysis indicated activation of the innate and adaptive immune responses. We found upregulation in phagocytosis and engulfment processes and B cell receptor signaling in both analyzed time-points (**Figure 2**). Moreover, we found an increase in the ribosome function, and biogenesis in MSC-treated mice, longitudinally (**Figure 2**). More precisely, the analysis of canonical pathways activation revealed the increase in IL-7 signaling, T and B cell signaling in the short- and long-term models compared to controls. Moreover, we found changes reflecting redox imbalance, such as an increase in HIF1α signaling and superoxide radicals degradation in the short-term model (**Figure 3A**). In addition to this observation, we noted the gradual downregulation in HIF1α signaling, IL-17 signaling, and B cell receptor signaling in the longer time point (**Figure 3A**). Furthermore, the analysis of the expression of genes clustered to the terms and processes related to airway inflammation, namely Th1-, Th2-, Th17-driven immune responses development, tight junction molecules, and mucins, revealed a relatively low number of significantly changed genes (**Figure 3B**), which stay in line with our *ex vivo* observations of effector T cells, epithelial barrier integrity, and histochemical staining’s.

### Pattern recognition receptors, macrophage activation, oxidative stress, and phagocytosis related genes are differentially expressed in the lungs after MSCs administration

**Figure 4:**
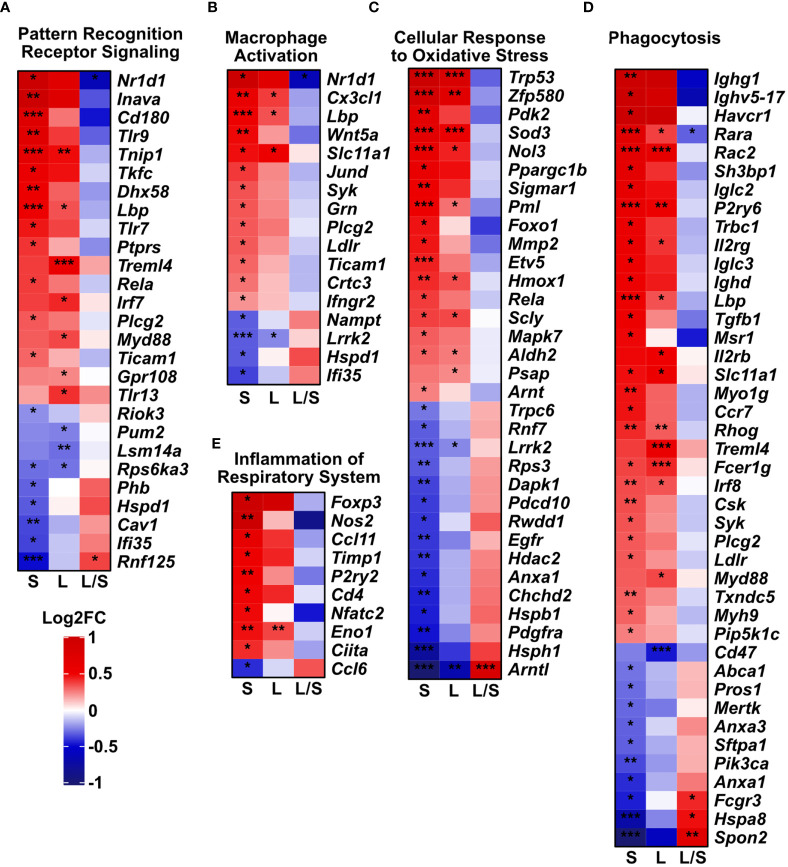
Innate immune gene clusters are differentially regulated upon mesenchymal stem cell administration. The analysis was performed based on Mouse Genome Informatics (MGI v.6.17) Gene Ontology Browser; http://www.informatics.jax.org/vocab/gene_ontology/; access 29^th^ June 2022). Only significant genes in either of the groups were plotted (adjusted p-value < 0.05). Heatmaps represent the changes in gene expression related to **(A)** Pattern Recognition Receptor Signaling Pathway (Biological Process; GO:0002221). **(B)** Macrophage Activation (Biological Process; GO:0042116). **(C)** Cellular Response to Oxidative Stress genes (Biological Process; GO:0034599). **(D)** Phagocytosis (with 4932438a13rik gene excluded due to unidentified biological role); (Biological Process; GO:0006909). **(E)** Inflammation of Respiratory System (genes predicated by functional analysis in Ingenuity Pathway Analysis software). S – short-term model; L – long-term model, L/S long- *vs* short-term model. Wald test with Benjamini-Hochberg correction was used; *p<0.05; **p<0.01; ***p<0.001.

Next, we analyzed deeper the expression profiles of genes related to oxidative stress and immune responses, macrophage activation and phagocytosis. We noted the dysregulated expression of pattern recognition receptors (PRRs, **Figure 4A**), macrophage activation (**Figure 4B**), oxidative stress (**Figure 4C**), phagocytosis (**Figure 4D**), and inflammation of the respiratory system (**Figure 4E**). Importantly, clustered genes present a trend to be downregulated in the latter time point towards the level observed in the untreated controls. However, in the short-term model, we noted an upregulated expression of most analyzed genes. However, a relatively low number of genes were significantly upregulated (FDR < 0.05) in analyzed models in the cluster reflecting inflammation within the respiratory system (9 upregulated genes among 50 defined in the cluster, **Figure 4E**).

### Commonly regulated genes form a low number of interactions

**Figure 5:**
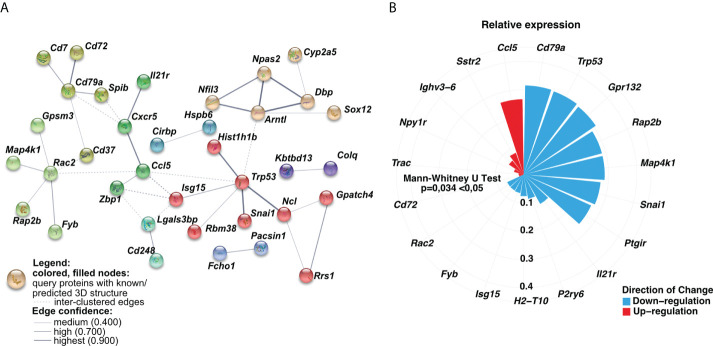
The relative expression of analyzed common genes changed longitudinally. **(A)** Genes-genes interaction networks corresponding to protein products were created using the String database. Nodes marked with the same color represent gene clusters. Solid lines show the connections within the individual cluster, whereas dashed lines refer to the interactions among the nodes. The line weight signifies the confidence of the relationship. **(B)** Circular bar plot indicated delta of genes expression with the altered z-scores at the time according to Ingenuity Pathway Analysis (IPA). The delta was obtained by subtraction between the expression of genes in the long-term and short-term models. The size and color of the bars represent the magnitude and the direction of change, respectively. A two-tailed Mann U Whitney test was performed to assess the statistical significance.

**Figure 6:**
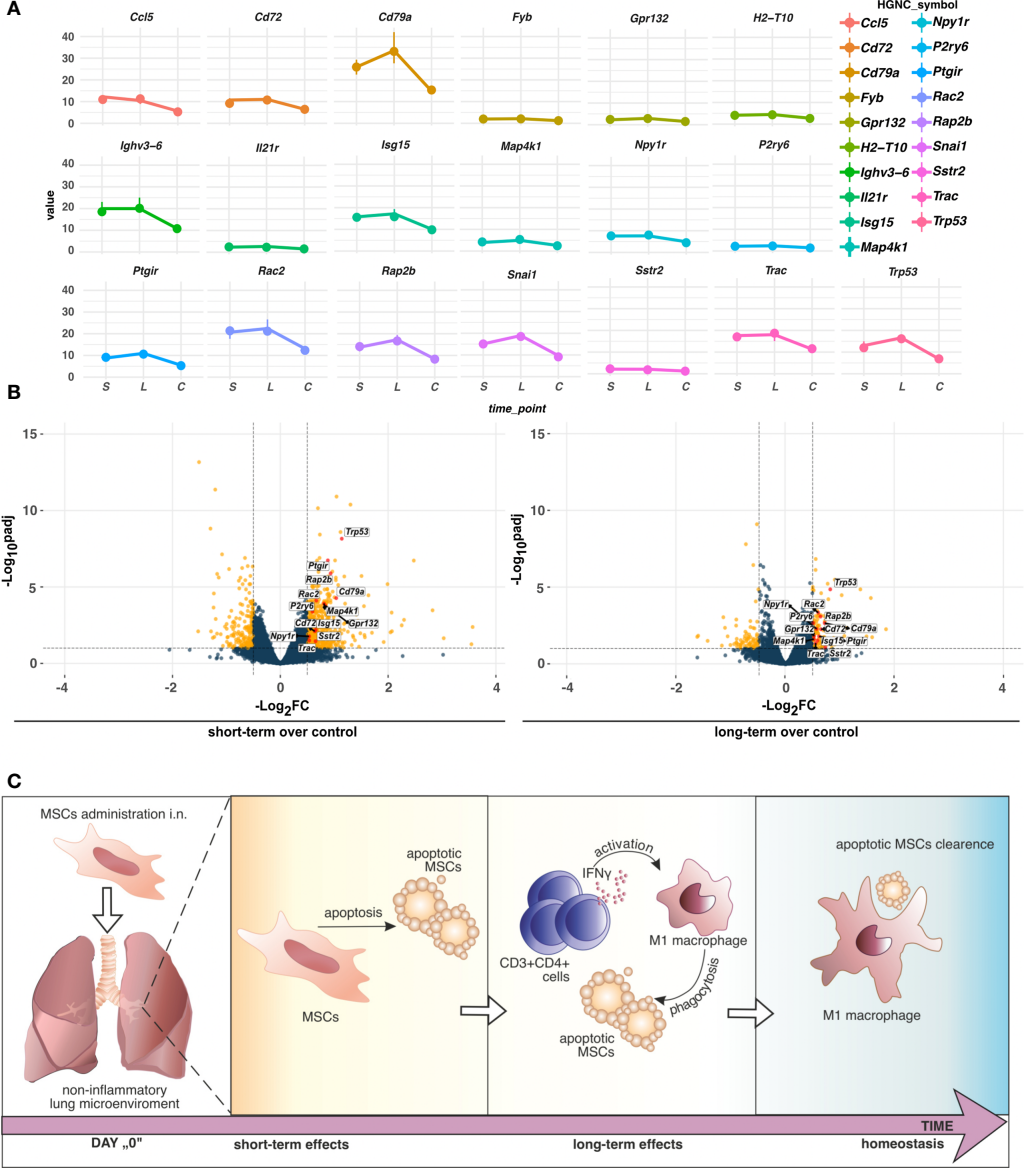
**(A)** TPM differences of genes altering the z-scores of pathways indicated by IPA. Dots represent the median (n=5) values of TPM in the group, short-term **(**S), long-term (L), or control **(C)**. Vertical lines connected to the dots indicate the upper (Q3) and lower (Q1) quantiles. **(B)** Distribution of the commonly regulated genes in short-term and long-term models. Commonly regulated genes in short- and long-term models altering the z-scores of pathways in IPA were marked red. Yellow dots refer to unique DEGs, whereas dark blue indicates no cutoff criteria met. Dashed horizontal and vertical lines represent the cutoffs for adjusted p-value (BH) < 0.1 and absolute Log2FoldChange > 0.5, respectively. **(C)** The proposed hypothesis of MSCs fate in the non-inflammatory microenvironment in the lungs. In non-inflamed lungs, MSCs undergo apoptosis which induces low-grade inflammation. An increase in IFNγ producing CD3^+^CD4^+^ cells activates phagocytic M1 macrophages to clear apoptotic MSCs.

Having found longitudinal changes in the gene expression profiles among MSCs-treated groups, next, we aimed to focus on the common genes for both short-term and long-term models. First, we found a low number of interactions among analyzed genes (**Figure 5**). The observed ones mainly reflect dysregulation of immune responses, namely dendritic cell, T cell, and B cell function *(Cd7, Cd37, Cd72, Cd79a, Spib, and Il-21r, Cxcr5, Ccl5, Zbp1)*. In addition, we observed interactions for ribosome biogenesis, function, and cell cycle (*Rrs1, Gpatch4, Trp35, Ncl, Rasl11a, Rbm38, Hist1h1b)*, shock proteins *(Cirbp, Hspb6)*, and circadian rhythm *(Dbp, Arntl, Npas2, Nfil3)* (**Figure 5A**). Furthermore, we analyzed the most significant genes with altered z-scores at the investigated time-points according to the Ingenuity Pathway Analysis. We found *Rap2b* relative expression as the one of most changed compared to the other delta expression of genes (**Figure 5B**). According to the Pathcards and Reactome database (https://pathcards.genecards.org; https://reactome.org; access date: 4^th^ July 2022), *Rap2b* is predicted to be involved in the neutrophils degranulation pathway, which is linked with the reactive oxygen species production (18). Moreover, we also noted a relatively relevant change in *Trp53* (**Figure 5B**), which is also recognized as an important contributor to oxidative stress-induced necrosis (19). Additionally, a significant change in *Gpr132* relative expression was observed, which is highly specific to infiltrating macrophages (20). Finally, we also observed a trend in a longitudinal decrease in the expression of the analyzed common genes (**Figures 6A, B**), which may indicate lung homeostasis reestablishment.

## Discussion

Despite significant progress in understanding MSCs biology and their therapeutic potential, MSC-based therapy approaches for respiratory tract diseases remain not available routinely (1). Initially, it was believed that MSCs can integrate within the airways (21–23). Currently, it became clear that MSC therapeutic potential is more related to their immunomodulatory functions exerted *via* direct cell-to-cell mediated interactions or even more by paracrine effects. In addition, major safety issues on their stability, long-term effects of application, and their fate were raised significantly delaying the implementation of MSCs-based therapies (2, 24–26). Here we demonstrated the short-term and long-term effects of human adipose tissue-derived MSC administration on non-inflamed healthy mice lungs. We showed an increased frequency of IFNγ-producing T cells and a simultaneous decrease in epithelial occludin and claudin 3 protein expression as a long-term effect of MSCs administration. In addition, we reported changes in the whole lung transcriptomic profiles indicating redox imbalance, hypoxia signaling pathway, and activation of macrophage’s phagocytic function. Our results indicate induction of low-grade inflammation as a long-term effect of MSCs transfer associated with graft clearance.

It was previously shown that allogenic MSCs may preserve within the airways for up to 72h (12, 27–29). However, more recently, Ferrini E *et al.*, by using third-generation of lentiviral vectors, showed that bone marrow-derived MSCs can be detected within the lung even longer than 14 days post intratracheal and intravenous administration (30). This observation explains the reported long-lasting beneficial effects of MSCs transfer to inflamed lungs. Therefore, in our study, we evaluated the short-term and long-term effects of MSCs administration.

As mentioned above, upon transplantation MSCs may undergo differentiation supporting regeneration or healing processes or, in response to inflammatory stimuli, may act as potent regulators of inflammation (31–33). Interestingly, MSCs apoptosis has been acknowledged as a mechanism of their immunosuppressive function and is believed to be required for their therapeutic effectiveness (33–36). Chang et al. demonstrated that apoptotic MSCs effectively downregulate inflammation, oxidative stress, and histopathological alternations in the lungs and kidneys in the mice sepsis model (36). These results remain consistent with “the dying stem cell hypothesis” introduced by Thum T. *et al.*, which shows modulation of the local immune responses by apoptosis of transplanted stem cells (33). However, in our model, MSCs were transferred to the non-inflamed lungs and we observed signs of low-grade inflammation as the long-term effects. We hypothesized that this effect might be associated with apoptotic graft or hetero-transplant clearance (37). This was additionally supported by observed transcriptional signatures in analyzed innate immune clusters, namely pattern recognition receptor signaling, macrophage activation, cellular response to oxidative stress, and phagocytosis. Our findings are partially in agreement with the recently published study by Preda MB et al., who proposed the *“hit and die”* concept indicating transplanted MSCs activate the hypoxia signaling pathway in the recipient organ, and subsequently undergo caspase-3/7 mediated apoptosis (35). In correspondence to the study, Galleu A *et al.* stated the hypothesis that cytokine-dependent priming is not required for the generation of apoptotic MSCs and induction of immunosuppression (31). Consequently, at the transplantation site, locally recruited macrophages remove apoptotic MSCs in the phagocytosis process and orchestrate anti-inflammatory responses (31, 36). Notably, IFNγ is a critical agent in the induction and activation of pro-inflammatory and highly phagocytic classically activated (M1 polarized) macrophages (38). These cells were shown previously to play a central role in the MSC graft removal (12, 35). Graft clearance may be associated with the cytotoxic effect of immune cells to the differentiating MSCs or be a consequence of MSCs apoptosis in response to proinflammatory stimulation, including IFNγ-mediated signaling (35). On the other hand, increased Th1 responses, associated with IFNγ release, affect epithelial barrier integrity by downregulation of the tight junction protein expression (39, 40). This explains observed in our study, decreased expression of occludin and claudin 3 in the long-term model. Furthermore, changes in the expression of analyzed clusters of genes in the latter time-point resemble the control pattern, which may indicate the gradual impairment of local low-grade inflammation induced upon MSCs apoptosis execution and graft removal.

## Conclusions

In summary, here, we showed short-term and long-term effects of *i.n.* administration of the MSCs to the non-inflamed lungs. Our results suggest that in the steady-state MSCs may undergo apoptosis in the non-inflammatory microenvironment. In turn, low-grade inflammation is induced in the late phases after MSC administration. Consequently, IFN-producing T cells may activate innate immune cells to efferocytosis, subsequently leading to the re-establishment of lung tissue homeostasis (**Figure 6C**). Thus, our results partially support “dying stem cells” and “hit and die” concepts. However, further studies are needed to fully understand the fate of MSCs within the lung microenvironment.

## Data availability statement

The datasets presented in this study can be found in online repositories. The names of the repository/repositories and accession number(s) can be found below: https://www.ncbi.nlm.nih.gov/geo/query/acc.cgi?acc=GSE200028.

## Ethics statement

The studies involving human participants were reviewed and approved by Ethical Committee at the Medical University of Bialystok. The patients/participants provided their written informed consent to participate in this study. The animal study was reviewed and approved by Local Ethical Committee in Olsztyn, Poland.

## Author contributions

Study design, AE and MM. Data collection, MT, MN, AZ, ASta, KG, NS, ASza, UK, AT, JR, MK, and AE. Data analysis and interpretation, MT, AJ, MN, AK, MK, and AE. Manuscript draft, MT, AJ, and AE. Supervision and coordination of the study, MM and AE. Review of the manuscript, CA, MS, MM, and AE. Data validation, MS, CA, MM, and AE. All authors contributed to the article and approved the submitted version.

## Funding

The publication was written during doctoral studies under the project № POWR.03.02.00-00-I050/16 co-funded from European Union funds, POWER 2014-2020. The article has been supported by the National Science Centre, Poland, Grant No. 2020/37/N/NZ5/04144. AJ and AZ were supported by the program “Best of the Best 4.0 (original name: Najlepsi z Najlepszych 4.0)” founded by the Ministry of Education and Science № N/POWER/21/001/1199. This study was conducted with the use of equipment purchased by the Medical University of Bialystok as part of the RPOWP 2007–2013 funding, Priority I, Axis 1.1, contract no. UDA- RPPD.01.01.00-20-001/15-00 dated 26.06.2015.

## Acknowledgments

All authors recognized Agnieszka Popielska MSc and the employees of the Center of Experimental Medicine for technical support.

## Conflict of interest

MT reports grant from National Science Centre, Poland, during the conduct of the study, grants from European Union funds, POWER 2014-2020, grants from National Centre for Research and Development, outside the submitted work. AJ and AZ reports grants and non-financial support from Ministry of Education and Science, Poland. CA reports research grants from the Swiss National Science Foundation, European Union (EU CURE), Novartis Research Institutes (Basel, Switzerland), Stanford University (Redwood City, Calif), and SciBase (Stockholm, Sweden), he is the Co-Chair for EAACI Guidelines on Environmental Science in Allergic diseases and Asthma, and serves on the Advisory Boards of Sanofi/Regeneron, Novartis, GlaxoSmithKline, and SciBase, and is the Editor-in-Chief of Allergy, outside the submitted work. MS reports grants from Swiss National Science Foundation, grants from GSK, grants from Novartis, personal fees from AstraZeneca, outside the submitted work. MM reports grants from National Centre for Research and Development, grant from Medical Research Agency, lecture fees from Astra Zeneca, Berlin-Chemie/Menarini, GSK, Takeda, Shire, Teva, Lek-Am, Celon, Sandoz, Pfizer, Hal Allergy, and had reimbursed conference costs and travel by Berlin-Chemie/Menarini, outside the submitted work. AE reports grant from National Science Centre, during the conduct of the study, grants from National Centre for Research and Development.

The remaining authors declare that the research was conducted in the absence of any commercial or financial relationships that could be construed as a potential conflict of interest.

## Publisher’s note

All claims expressed in this article are solely those of the authors and do not necessarily represent those of their affiliated organizations, or those of the publisher, the editors and the reviewers. Any product that may be evaluated in this article, or claim that may be made by its manufacturer, is not guaranteed or endorsed by the publisher.
