# Supplementary material for: The short-term and long-term effects of intranasal mesenchymal stem cell administration to noninflamed mice lung
Source: Front Immunol. 2022 Sep 16;13:967487. doi: 10.3389/fimmu.2022.967487 (PMC9523259; doi:10.3389/fimmu.2022.967487)
Supplement: Supplementary file 1 [file DataSheet_1.pdf]

The short-term and long-term effects of intranasal mesenchymal stem cell administration to noninflamed mice lung

Marlena Tynecka[1], Adrian Janucik[1], Magdalena Niemira[2], Arkadiusz Zbikowski[3], Nino Stocker[4], Agnieszka Tarasik[5], Aleksandra Starosz[1], Kamil Grubczak[1], Anna Szalkowska[2], Urszula Korotko[6], Joanna Reszec[5], Miroslaw Kwasniewski[6], Adam Kretowski[2,7], Cezmi Akdis[4], Milena Sokolowska[4], Marcin Moniuszko[1,8,*,†], Andrzej Eljaszewicz[1,*,†]

[1]Department of Regenerative Medicine and Immune Regulation, Medical University of Bialystok, Bialystok, Poland

[2]Clinical Research Centre, Medical University of Białystok, Bialystok, Poland

[3]Department of Medical Biology, Medical University of Bialystok, Bialystok, Poland

[4]Swiss Institute of Allergy and Asthma Research, University of Zurich, Davos, Switzerland

[5]Department of Medical Pathomorphology, Medical University of Bialystok, Bialystok, Poland

[6]Centre for Bioinformatics and Data Analysis, Medical University of Bialystok, Bialystok, Poland

[7]Department of Endocrinology, Diabetology and Internal Medicine, Medical University of Bialystok, Bialystok, Poland

[8]Department of Allergology and Internal Medicine, Medical University of Bialystok, Bialystok, Poland

[†]These authors share senior authorship.

* Correspondence:

Andrzej Eljaszewicz, PhD

Department of Regenerative Medicine and Immune Regulation

Medical University of Bialystok, Bialystok, Poland

Ul. Waszyngtona 13, 15-269 Bialystok, Poland

Email: andrzej.eljaszewicz@umb.edu.pl

Prof. Marcin Moniuszko, MD PhD

Department of Regenerative Medicine and Immune Regulation

Medical University of Bialystok,

Ul. Waszyngtona 13, 15-269 Bialystok, Poland

Department of Allergology and Internal

Medicine, Medical University of Bialystok,

Ul. M. Sklodowskiej-Curie 24a, Bialystok, 15-276 Bialystok,

Poland Email: marcin.moniuszko@umb.edu.pl

Supplementary materials and methods

Adipose tissue-derived mesenchymal stem cells isolation and culture

Mesenchymal stem cells were isolated from adipose tissue obtained from the bariatric patients (n=5) admitted to the 1st Clinical Department of General and Endocrinology Surgery, Medical University of Bialystok. Harvested adipose tissue was incubated with collagenase IV (Gibco) in standard cell culture conditions (37°C, 5% CO2) for 45 minutes followed by inactivation in 10% fetal bovine serum (FBS, PAN Biotech) in Dulbecco's Modified Eagle's Medium (DMEM, PAN Biotech). Next adipose tissue was filtered through strainers (100µM pore size) and centrifuged at 400g for 5 minutes. Isolated cells were cultured in Mesenchymal Stem Cells Basal Medium (ATCC) supplemented with Mesenchymal Stem Cell Growth Kit for Adipose, Umbilical, and Bone Marrow-derived MSCs (ATCC) up to 3rd passage. Isolated adipose tissue-derived MSCs fulfill the following minimal criteria proposed by the International Society for Cellular Therapy: 1) adhere to the plastic surface in cell culture; 2) express at least CD73, CD90, and CD105, while they are negative for CD45 and HLA-DR; 3) differentiate into adipocytes, osteocytes, and chondrocytes. Expanded adipose tissue-derived MSCs cells were subjected to the experimental mice model (500 000 cells/transfer, with viability higher than 95%). Cell viability and density were assessed by using trypan blue in the Burker chamber by two independent scientists.

Adipose tissue-derived MSCs characterization in vitro

The phenotype and multipotency properties of isolated MSCs were confirmed prior to implementation in the experimental mice model using the following methods.

- *Flow cytometry analysis of adipose tissue-derived MSCs phenotype*

To confirm the specific phenotype of MSCs, isolated cells were incubated with monoclonal antibodies conjugated with fluorochrome according to standard protocol. For staining, appropriate FMO control was performed. The antibodies used for MSCs phenotype confirmation were listed in Table S1. Samples were acquired using FACSCalibur (Becton Dickinson) and analyzed with FlowJo v.10 (BD Biosciences).

Supplementary Table S1. Antibodies used to confirm the adipose tissue-derived MSCs phenotype.

ANTIBODIES	CLONE	SOURCE	IDENTIFIER
anti-human CD73 FITC	AD2	BioLegend	Cat. 344016
anti-human CD90 PE	5E10	BioLegend	Cat. 328110
anti-human CD105 APC	43A3	BioLegend	Cat. 323208
anti-human CD45 PerCP	2D1	BioLegend	Cat. 368506
anti- human HLA-DR APC	TU36	BD Pharmingen	Cat. 559868

- *Assessment of multipotency of adipose tissue-derived MSCs*

Differentiation of adipose tissue-derived MSCs to mesodermal lineage cells was verified using the Human Mesenchymal Stem Cell Functional Identification Kit (R&D Systems, Cat. SC006) according to the provided protocol. MSCs were incubated in the dedicated adipocytes, osteocytes, and chondrocytes differentiation media for up to 21 days. To analyze the differentiation into three derivatives, specific markers provided in the kit were used: adipocyte marker (Goat Anti-Mouse FABP4 Antigen Affinity-purified Polyclonal Antibody), osteocyte marker (Mouse Anti-Human Osteocalcin Monoclonal Antibody), and chondrocyte marker (Goat Anti-Human Aggrecan Antigen Affinity-purified Polyclonal Antibody). Adipocytes, osteocytes, and chondrocytes specimens were acquired using Olympus FV1200 confocal microscope (Olympus).

Supplementary Table S2. Antibodies used in the study

ANTIBODIES	CLONE	SOURCE	IDENTIFIER
**Flow cytometry staining**			
anti-mouse CD45 PE	30-F11	Biolegend	Cat. 103106
anti-mouse CD3e	145-2C11	Biolegend	Cat. 100320
anti-mouse CD4 PE/Dazzle 594	RM4-5	Biolegend	Cat. 100566
anti-mouse IFNg PerCP-Cy5.5	XMG1.2	eBioscence	Cat.45-7311-80
anti-mouse IL-4 APC	11B11	eBioscence	Cat.17-7041-82
anti-mouse IL-17A Alexa Fluor 488	eBio17B7	eBioscence	Cat. 53-7177-81
anti-mouse IL-10 APC/Cy7	JES5-16E3	Biolegend	Cat. 505036
**Immunocytochemistry (ICC) staining**			
polyclonal rabbit anti- ZO-1	-	Invitrogen	Cat. 40-2200
monoclonal mouse anti- Occludin	OC-3F10	Invitrogen	Cat. 33-1500
polyclonal rabbit anti-Claudin 3	-	Invitrogen	Cat. 34-1700
goat anti-mouse IgG (H+L) secondary Alexa Fluor 488	-	Invitrogen	Cat. A-11001
goat anti-rabbit IgG (H+L) secondary Alexa Fluor 488	-	Invitrogen	Cat. A-11008

Supplementary Table S3 List of excluded RIKEN genes from the transcriptomic analysis considering commonly regulated genes in the short- and long-term group.

Ensembl	HGNC
ENSMUSG00000028536	2610528J11Rik
ENSMUSG00000043168	4930426D05Rik
ENSMUSG00000062319	Gm10115
ENSMUSG00000071540	3425401B19Rik
ENSMUSG00000075514	Gm13375
ENSMUSG00000075538	Gm10855
ENSMUSG00000097194	9330175E14Rik
ENSMUSG00000097296	Gm26532
ENSMUSG00000101365	Gm19325
ENSMUSG00000101693	Gm19461
ENSMUSG00000102142	Gm26930
ENSMUSG00000104476	Gm38211
ENSMUSG00000105272	Gm43071
ENSMUSG00000105771	2900064K03Rik
ENSMUSG00000105985	Gm42993
ENSMUSG00000106237	Gm8066
ENSMUSG00000107290	Gm43282
ENSMUSG00000108046	Gm43924
ENSMUSG00000108443	Gm44510
ENSMUSG00000108456	4732496C06Rik
ENSMUSG00000109157	Gm44829
ENSMUSG00000109363	Gm44668
ENSMUSG00000112307	Gm48751
ENSMUSG00000114501	Gm48582
ENSMUSG00000114608	Gm36161
ENSMUSG00000117729	Gm5242

Supplementary figures

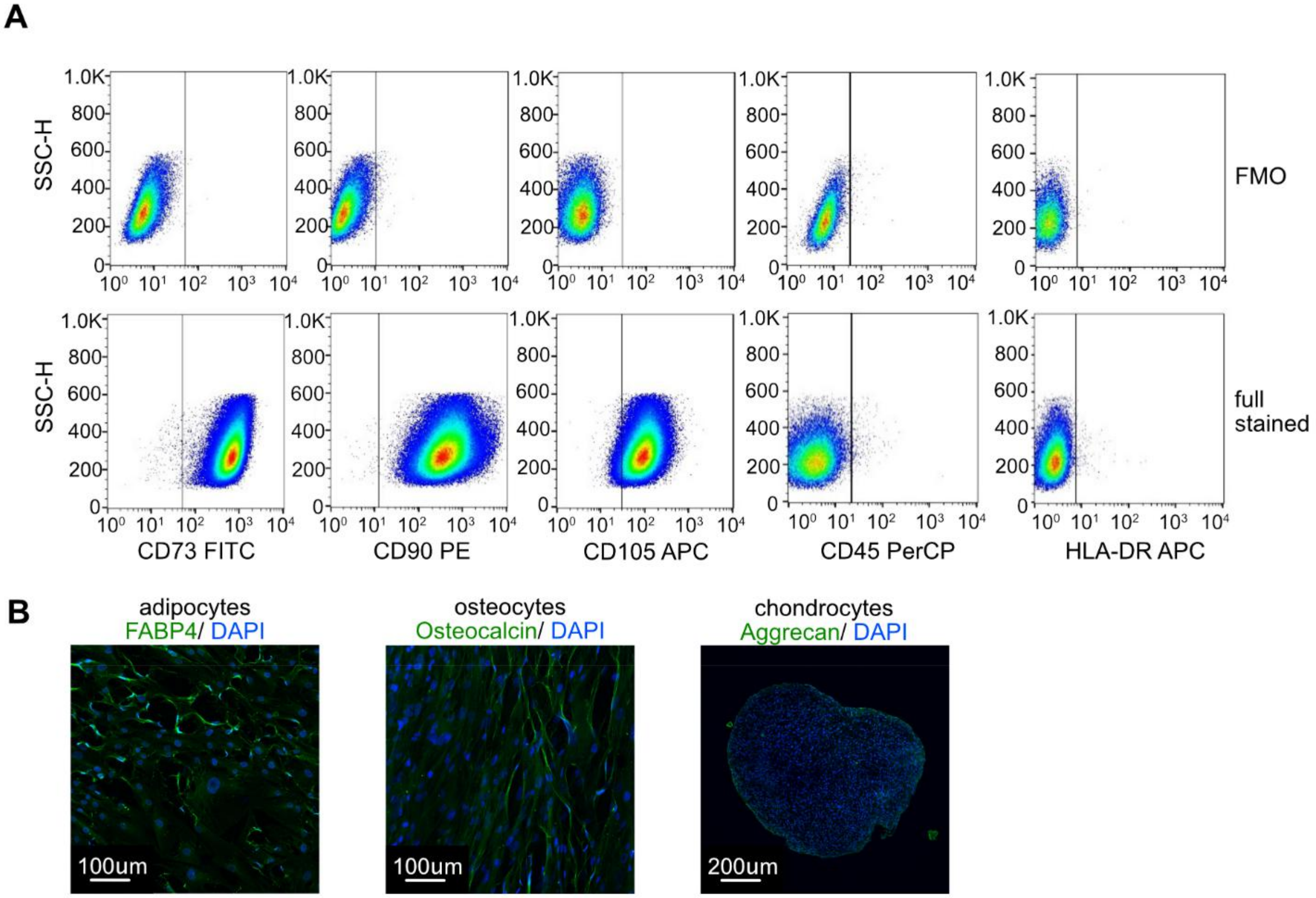

Supplementary Figure S1. Adipose tissue-derived mesenchymal stem cells A. phenotype confirmation and B. multipotency verification. A. Adipose tissue-derived MSCs were characterized by the expression of CD73, CD90, and CD105 markers, while they are negative for CD45 and HLA-DR (n=5). **B.** Representative photos of confocal staining of adipose tissue-derived MSCs differentiated to mesodermal lineage cells. Cells were visualized using specific antibodies, namely FABP4 (adipocyte marker), osteocalcin (osteocytes marker), and aggrecan (chondrocyte marker); DAPI- blue, nucleus; Alexa Fluor 488- green, positive signal of analyzed markers.

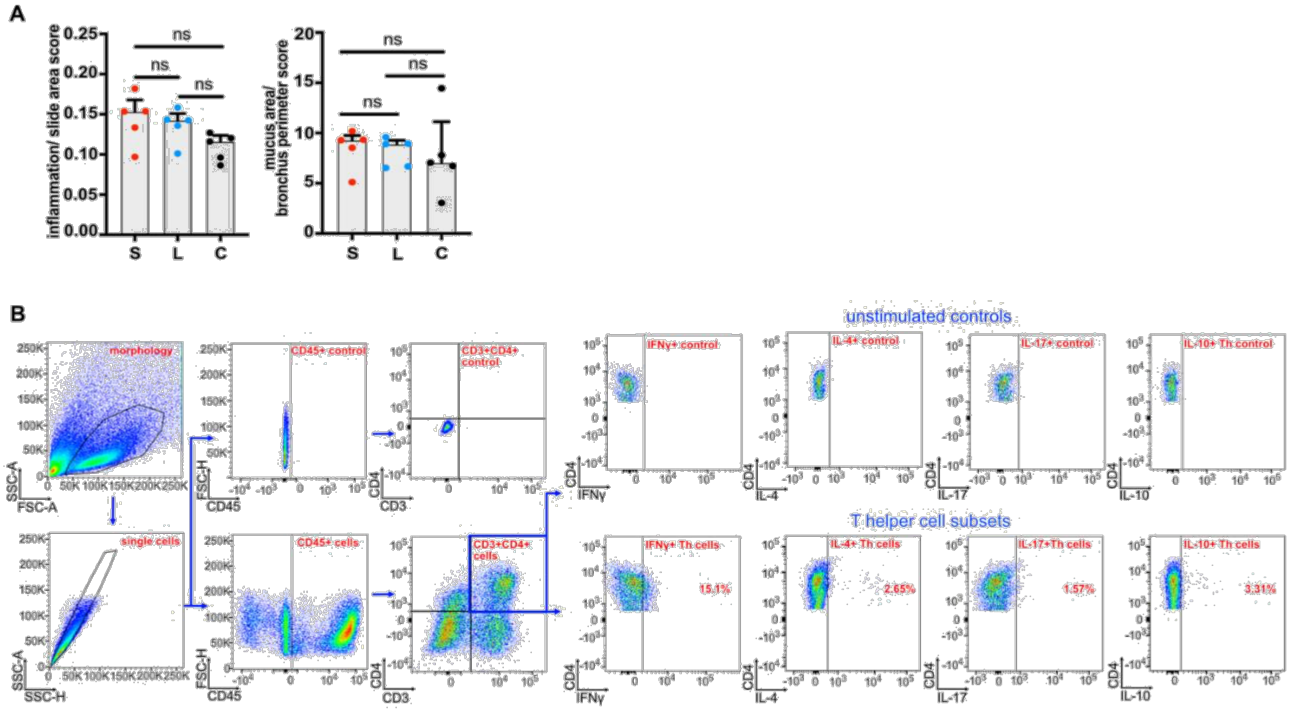

Supplementary Figure S2. A. Summary of quantification results of hematoxylin & eosin (upper bar plot) and periodic acid-shiff staining (lower barplot). S – short-term model; L – long-term model; C – control; U Mann-Whitney test was used to assess the differences among investigated groups; $p<0.05$ was considered statistically significant; ns – not significant; n= 5 **B. Representative gating strategy of IFNg, IL-4, IL-17, or IL-10 producing CD3+CD4+ T cell.** The cells were gated according to forward (FSC), and side (SSC) scatter (morphology) followed by doublets discrimination (single cells). Next, CD45- cells were excluded from the analysis (CD45+ cells), and CD3+CD4+ cells were gated (CD3+CD4+ cells). Finally, cytokine-producing cells were gated according to the unstimulated control. FlowJo v.10 software was used for data analysis.
